# Identification of the presence of ischaemic stroke lesions by means of texture analysis on brain magnetic resonance images

**DOI:** 10.1016/j.compmedimag.2019.02.006

**Published:** 2019-06

**Authors:** Rafael Ortiz-Ramón, Maria del C. Valdés Hernández, Victor González-Castro, Stephen Makin, Paul A. Armitage, Benjamin S. Aribisala, Mark E. Bastin, Ian J. Deary, Joanna M. Wardlaw, David Moratal

**Affiliations:** a Centre for Biomaterials and Tissue Engineering, Universitat Politècnica de València, Valencia, Spain; b Department of Neuroimaging Sciences, Centre for Clinical Brain Sciences, University of Edinburgh, Edinburgh, UK; c Department of Electric Systems and Automatics Engineering, Universidad de León, León, Spain; d Department of Cardiovascular Sciences, University of Sheffield, Sheffield, UK; e Department of Psychology, University of Edinburgh, Edinburgh, UK; f Department of Computer Science, Lagos State University, Lagos, Nigeria; g Centre for Cognitive Ageing and Cognitive Epidemiology, University of Edinburgh, Edinburgh, UK

**Keywords:** Texture analysis, Radiomics, Stroke, Small vessel disease, White matter hyperintensities

## Abstract

**Background:**

The differential quantification of brain atrophy, white matter hyperintensities (WMH) and stroke lesions is important in studies of stroke and dementia. However, the presence of stroke lesions is usually overlooked by automatic neuroimage processing methods and the-state-of-the-art deep learning schemes, which lack sufficient annotated data. We explore the use of radiomics in identifying whether a brain magnetic resonance imaging (MRI) scan belongs to an individual that had a stroke or not.

**Materials and methods:**

We used 1800 3D sets of MRI data from three prospective studies: one of stroke mechanisms and two of cognitive ageing, evaluated 114 textural features in WMH, cerebrospinal fluid, deep grey and normal-appearing white matter, and attempted to classify the scans using a random forest and support vector machine classifiers with and without feature selection. We evaluated the discriminatory power of each feature independently in each population and corrected the result against Type 1 errors. We also evaluated the influence of clinical parameters in the classification results.

**Results:**

Subtypes of ischaemic strokes (i.e. lacunar vs. cortical) cannot be discerned using radiomics, but the presence of a stroke-type lesion can be ascertained with accuracies ranging from 0.7 < AUC < 0.83. Feature selection, tissue type, stroke subtype and MRI sequence did not seem to determine the classification results. From all clinical variables evaluated, age correlated with the proportion of images classified correctly using either different or the same descriptors (Pearson r = 0.31 and 0.39 respectively, p < 0.001).

**Conclusions:**

Texture features in conventionally automatically segmented tissues may help in the identification of the presence of previous stroke lesions on an MRI scan, and should be taken into account in transfer learning strategies of the-state-of-the-art deep learning schemes.

## Highlights

• Radiomics in conventionally segmented tissues can identify MRI scans that had a stroke.
• Patient’s advanced age can negatively influence classification results.
• Feature selection and stroke subtype influence but do not determine accuracy.
• Stroke subtype cannot be ascertained from texture analysis in brain tissues.

## 1 Introduction

### 1.1 Importance of automatically detecting the presence/absence of a previous stroke

The differential quantification of brain atrophy, white matter hyperintensities (WMH) and stroke lesions (i.e. either acute or old, symptomatic or asymptomatic) is important in studies of stroke and dementia. However, stroke lesions, in magnetic resonance images (MRI), can have similar signal intensities as WMH and cerebrospinal fluid (CSF) (i.e. a proxy for brain atrophy), and may be accidentally considered WMH or CSF by image processing methods. Disentangling the effect that each of them has in cognitive and health indicators is crucial for individual prognosis of stroke outcomes and for understanding the pathophysiology of stroke and ageing. For example, although the most serious consequence of a stroke is neuronal death, indicators of neurofibrillary degeneration have been associated with 1-year post-stroke brain atrophy (Ihle-Hansen et al., 2017), assessed from MRI. Brain tissue atrophy and stroke lesion volume, but not WMH volume have been cited as neuroimaging determinants of post-stroke cognitive performance (Puy et al., 2018). On the other hand, there is evidence that WMH and not old stroke lesion volume is associated with brain atrophy and cognitive decline in normal ageing (Habes et al., 2016; Valdés-Hernández et al., 2013).

The differential assessment of these imaging markers (i.e. WMH, stroke lesion and brain atrophy) is also necessary in the design of clinical trials that use these parameters as outcome measurements, for estimating the sample size. The latter is driven by a measure of “uncertainty” in the estimation of the outcome measurements, as well as the absolute difference between these measurements in the groups of individuals involved in the trials. A study showed that failure to exclude stroke lesion volume in the volume reported as WMH added an uncertainty of 20%, which could cause an increase in the sample size calculation and consequently impact in the trial duration and cost (Wang et al., 2012). Adjudicating treatment response to an increase/decrease in WMH instead of stroke lesion due to miscalculating their true volumes potentially could miss effective treatments or make ineffective treatments look as if they were beneficial. The same study found that for individual patients, failing to consider the tissue loss due to stroke when measuring brain atrophy could increase the apparent brain volume loss by up to 21.25 ml more, representing up to 4 times more atrophy than the true value.

### 1.2 The-state-of-the-art neuroimaging methods for recognising a stroke

Neuroimaging studies of stroke benefit from the use of diffusion weighted images, which are part of routine clinical stroke neuroimaging protocols as they facilitate the identification of stroke lesions. However, it has been reported that these images do not identify the presence of stroke on approximately a third of patients seen in clinics with a non-disabling stroke (Makin et al., 2015). Also, they are not always part of the neuroimaging protocol for studies of ageing and dementia. In the last few decades, the increase in computational power of affordable computer platforms has given a boost in the application of machine-learning methods to medical image analysis. A recent review on machine learning methods applied only to the study of dementia using MRI (Pellegrini et al., 2018), found 5747 studies, excluding reviews and animal studies, published up to January 2016. From 2016 onwards, deep learning methods, principally convolutional neural networks (CNN), have dominated advances in this field (Litjens et al., 2017; Shen et al., 2017). However, the requirements of still expensive hardware and a large amount of annotated data have limited their applicability, favouring the application of the more conventional ones (Giger, 2018). Moreover, CNN, the deep learning method by excellence in Computer Vision, suffer a considerable loss in performance in the following scenarios: 1) in the presence of pathologies that differ from those in the training set, 2) with datasets acquired with different imaging protocols, or using different sequences (i.e. the task domain changes), 3) when performing tasks that are related to but not the same as that on which they were trained (e.g. lesion segmentation vs. lesion assessment) (Rachmadi et al., 2018). To overcome these limitations, there are several ways to enhance the performance of CNN architectures without modifying the architecture itself. These are known as “enhanced learning techniques” and comprise: 1) modifying the input data by adding information derived from internal and external sources (i.e. data augmentation), and/or 2) re-purposing a model trained for one task to perform a second related task (i.e. transfer learning). For these techniques to be successful, it is important to understand which imaging features are relevant for the machine and integrate this “domain” knowledge in (re)training the CNN (Liu et al., 2018).

### 1.3 Suitability of radiomics for identifying the presence/absence of a previous stroke

Radiomics is a promising field that aims to improve precision in diagnosis, assess prognosis or predict treatment response by extracting a large number of quantitative descriptors from medical imaging data. Radiomics practice is based on the hypothesis that medical images contain valuable information at the tissue/organ level (macroscopic level) that reflects the underlying pathophysiology of the tissue (microscopic level) and that these relationships can be revealed by means of quantitative imaging features (Gillies et al., 2016; Lambin et al., 2012, 2017). Texture features are the imaging features most widely used in radiomics since they have proved to be efficient in describing the voxel interrelationships and the grey level distributions within images, allowing quantification of the intrinsic heterogeneity (vs. homogeneity) that may not be visually perceived, thus facilitating the characterisation and classification of different tissues (Castellano et al., 2004). These parameters can be correlated or combined with other medical information such as demographic, clinical, histologic or genomic data in order to improve decision-making tasks (Avanzo et al., 2017; Kumar et al., 2012; Larue et al., 2017). Texture analysis can be applied to different/multiple imaging modalities, and the selection of the appropriate technique to investigate each disease or lesion depends on several factors. Recently, MRI has increased in popularity in radiomics studies due to its growing incorporation into routine clinical practice and its ability to produce high-quality images (Larroza et al., 2016). Specifically for the study of SVD and stroke lesions, several studies that used MRI have successfully applied texture analysis to different tasks (González-Castro et al., 2017; Kassner et al., 2009; Leite et al., 2015; Valdés-Hernández et al., 2017; Viksne et al., 2015). One of them found that texture in normal-appearing tissues showed promise for stratifying patients according to their SVD and WMH burden (Valdés-Hernández et al., 2017).

### 1.4 Study rationale, hypotheses and research questions

As normal-appearing tissue can be assessed using well-established automatic image processing tools (e.g. SPM (https://www.fil.ion.ucl.ac.uk/spm/), FSL (https://fsl.fmrib.ox.ac.uk/fsl/fslwiki/), FreeSurfer (https://surfer.nmr.mgh.harvard.edu/), BrainVisa (http://brainvisa.info/web/index.html) to mention just a few) with a high level of accuracy, and given the effect that a stroke is known to have not only in the affected region, but also in unaffected tissue, we investigated the feasibility of using texture analysis in the tissues conventionally identified in structural MR images to identify the presence/absence of a previous stroke. Moreover, as WMH (i.e., a confound for the automatic identification of ischaemic stroke lesions) have been defined as having a mainly vascular origin (Wardlaw et al., 2013), we also analysed whether texture in WMH can help increase the likelihood of accurately identifying the presence of a major ischaemic stroke lesion. We therefore hypothesised that a conventional classifier that uses textural features in WMH and tissues commonly segmented by current automatic image processing techniques, can discriminate the brain MRI of individuals that had a stroke from those who had not, so as a post-processing step of removing the stroke effect could be done, although the type of stroke (i.e. cortical vs. lacunar) might be difficult to be ascertained. Specifically, our research questions are: 1) Can a texture-based automatic classifier discriminate a routine clinical structural brain MRI scan of a patient with a recent lacunar stroke from a brain MRI scan from a patient with a recent mild cortical stroke? 2) Can a texture-based automatic classifier discriminate between the MRI data of an individual who had a previous stroke from an individual of similar age who never had a stroke? We also investigated the effect of age in the classification.

## 2 Materials and methods

### 2.1 Subjects

To answer our two research questions we used MRI data from individuals enrolled in two different prospective studies: one study of stroke mechanisms (Wardlaw et al., 2017) and one study of cognitive ageing (Taylor et al., 2018). The sample from the first study included MRI data from 100 patients (54 women and 46 men, mean age 65.3 years old, SD 11 years) which had a lacunar (n = 50) or mild (i.e. mRS <3) cortical (n = 50) ischaemic stroke less than 2 weeks prior to the MRI scan (i.e. post-acute stage). The sample from the second study included MRI data from 100 individuals from a year-of-birth cohort (53 women and 47 men, mean age 73.2 years old, SD 0.6 years) who were either stroke free (n = 50) or had a prior ischaemic stroke in the non-acute (i.e. chronic) phase identifiable on imaging (n = 50). The data selection was performed randomly and fully automatically, only taking account that the four subgroups (i.e. 1) recent lacunar stroke, 2) recent cortical stroke, 3) no stroke, 4) old stroke) were equal-sized. To evaluate the influence of age in the classification of scans having a stroke or not, we used brain MRI data from 36 individuals from another year-of-birth cohort. They were also enrolled in a study of cognitive ageing (Taylor et al., 2018) (20 women and 16 men, mean age 91 SD 0.5 years). From this sample, 22/36 individuals never had a stroke (i.e. at least identifiable in imaging).

All studies that provided data and involved human participants were conducted in accordance with the 1964 Helsinki declaration and its later amendments, with protocols and ethical standards approved by the following Scottish Research Ethics Committees: Lothian Research Ethics Committee (09/S1101/54, LREC/2003/2/29, REC 09/81101/54), the NHS Lothian R + D Office (2009/W/NEU/14), and the Multi-Centre Research Ethics Committee for Scotland (MREC/01/0/56) (Valdés-Hernández et al., 2015a,b; Wardlaw et al., 2017).

### 2.2 Imaging protocol

All brain MRI data were acquired on a 1.5 T GE Signa LX clinical scanner (General Electric, Milwaukee, WI), equipped with a self-shielding gradient set and manufacturer supplied eight-channel-phased array heal coil. The MRI acquisition protocols of the studies that provided data for these analyses differed. The MRI sequences considered in this work were 3D T1-weighted (T1W) inversion recovery-prepared spoiled gradient echo (SPGR), axial 2D T2-weighted (T2W) and axial 2D fluid-attenuated inversion recovery (FLAIR) brain images. For the stroke study, the T1W had TR/TE/TI = 7.3/2.9/500 ms, 8° flip angle, 33 × 21.5 cm^2^ field of view (FoV), 256 × 146 acquisition matrix, 100 × 1.8 mm slices, the T2W sequence had a propeller acquisition with TR/TE = 6000/90 ms, 24 × 24 cm FoV, 384 × 384 acquisition matrix, 28 × 5 mm slices, 1 mm slice gap, and the FLAIR had TR/TE/TI = 9000/153/2200 ms, 24 × 24 cm FoV, 384 × 224 acquisition matrix, 28 × 5 mm slices, 1 mm slice gap. Both year-of-birth cohort (normal ageing) studies had the same MRI acquisition protocol: T1W had TR/TE/TI = 9.7/3.984/500 ms, 25.6 × 25.6 cm^2^ FoV, 192 × 192 acquisition matrix, voxel size 1 × 1 × 1.3 mm^3^, T2W had TR/TE = 11320/102 ms, 25.6 × 25.6 FoV, 256 × 256 acquisition matrix, 80 × 2 mm slices, no inter-slice gap; and FLAIR had TR/TE/TI = 9000/140/2200 ms, 256 × 192 acquisition matrix, and 40 × 4 mm slices. All acquisitions were zero-filled and resampled to either 256 × 256 or 512 × 512 in-plane resolution matrices.

### 2.3 Image processing

**Fig. 1:**
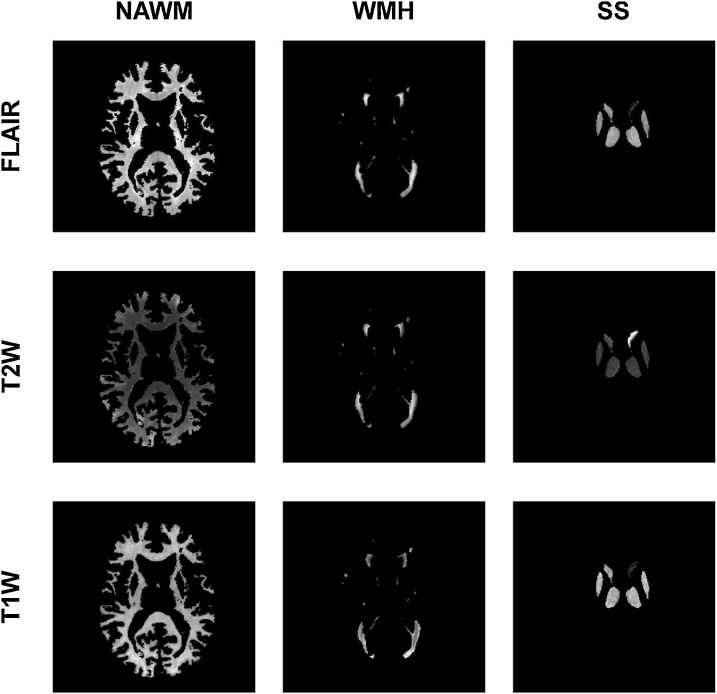
Set of images obtained in each patient. In this representative case, T1-weighted (T1W), T2-weighted (T2W) and fluid-attenuated inversion recovery (FLAIR) brain images of normal appearing white matter (NAWM), white matter hyperintensities (WMH) and subcortical structures (SS) of a lacunar stroke patient are presented.

The segmentation of the brain tissues and structures was performed following the protocol described in (Valdés-Hernández et al., 2015a,b). Briefly, binary masks of normal appearing white matter (NAWM) and WMH were obtained using a multispectral segmentation method (Valdés-Hernández et al., 2010) (www.sourceforge.com/projects/bric1936) followed by manual editing to correct for possible errors. The structures of the basal ganglia and thalami were fully automatically extracted using a combination of three tools from the FMRIB software library (FSL) (https://fsl.fmrib.ox.ac.uk/fsl/fslwiki) (Jenkinson et al., 2012): Smallest Univalue Segment Assimilating Nucleus (SUSAN), FMRIB's Linear Image Registration Tool (FLIRT) and a model-based segmentation/registration tool (FIRST), combined on an automatic pipeline developed in-house, and also manually corrected if necessary. Binary masks of NAWM, WMH and subcortical structures were mapped into the T1W, T2W and FLAIR sequences as illustrated in Fig. 1

### 2.4 3D texture analysis

**Fig. 2:**
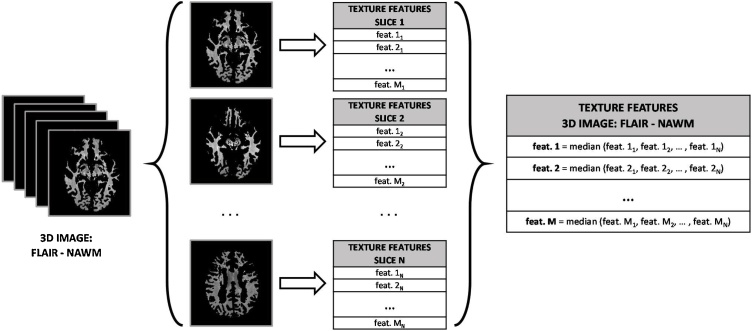
Process followed to extract the 3D features of a FLAIR image of the NAWM. The same process is applied to all the images of each MRI modality (FLAIR, T1W and T2W) and of each tissue/structure (NAWM, WMH and SS).

Textural features were extracted from a total of 1800 3D sets of images (2 prospective studies × 100 individual data × 3 MRI sequences × 3 brain tissues/structures). A simple approach to capture the volumetric information of each 3D image was implemented: we first extracted the 2D texture features from each slice of each 3D image, and then the 3D texture features of the image were obtained by computing the median of the values of all the slices. This process is illustrated in Fig. 2. Using this approach, the grey-level distributions in the third dimension are not considered; however, it has been shown that features computed with this 2D averaging method are more discriminative than features extracted from a single slice (Larroza et al., 2016). Additionally, all features were standardized to zero mean and unit variance to improve numerical stability in the model training process. Also, zero-variance and near-zero-variance predictors were removed for the same reason (Kuhn and Johnson, 2013b). Finally, some features failed to give a valid numeric value for some patients (e.g. while attempting to be calculated on small WMH clusters), so these features were also removed to avoid computational problems in the training process.

The feature extraction process was performed in MATLAB (R2015b; The MathWorks Inc., Natick, MA, USA) taking as a reference the code implemented in (Alegre et al., 2012).

### 2.5 Texture descriptors

A total of 114 features were extracted from each of the 1800 3D images and grouped into five different sets of textural features according to the texture analysis method employed: Grey-level Co-occurrence Matrix features (GLCM: 13 parameters), Grey-Level Run-Length Matrix features (GLRLM: 11 parameters), Local Binary Patterns features (LBP: 40 parameters), Wavelet Statistical features (WSF: 26 parameters) and Wavelet Co-occurrence features (WCF: 24 parameters). Table 1 shows all textural features extracted from each method.

**Table 1:** Texture features extracted.

Method	Textural Features	Number of Features
GLCM	Energy, Contrast, Correlation, Variance, Homogeneity, Sum average, Sum variance, Sum entropy, Entropy, Difference variance, Difference entropy, First information measure of correlation (FIMC), Second information measure of correlation (SIMC)	13
GLRLM	Short Run Emphasis (SRE), Long Run Emphasis (LRE), Grey-level Non-uniformity (GLN), Run-Length Non-uniformity (RLN), Run Percentage (RP), Low Grey-level Run Emphasis (LGRE), High Grey-level Run Emphasis (HGRE), Short Run Low Grey-level Emphasis (SRLGE), Short Run High Grey-level Emphasis (SRHGE), Long Run Low Grey-level Emphasis (LRLGE), Long Run High Grey-level Emphasis (LRHGE)	11
LBP	LBP histogram bins: LBP_1_, LBP_2_, LBP_3_, …, LBP_36_	40
	LBP image statistics: LBP_Median, LBP_Variance, LBP_Skewness, LBP_Kurtosis	
WSF	Mean_OI, SD_OI (OI: Original image)	26
	Mean_LL*_i_*, Mean_LH*_i_*, Mean_HL*_i_* and Mean_HH*_i_*, for *i* = 1, 2, 3	
	SD_LL*_i_*, SD_LH*_i_*, SD_HL*_i_* and SD_HH*_i_*, for *i* = 1, 2, 3	
WCF	Energy_LL_1_, Contrast_LL_1_, Correlation_LL_1_, Homogeneity_LL_1_, Entropy_LL_1_, Variance_LL_1_	24
	Energy_LH_1_, Contrast_LH_1_, Correlation_LH_1_, Homogeneity_LH_1_, Entropy_LH_1_, Variance_LH_1_	
	Energy_HL_1_, Contrast_HL_1_, Correlation_HL_1_, Homogeneity_HL_1_, Entropy_HL_1_, Variance_HL_1_	
	Energy_HH_1_, Contrast_HH_1_, Correlation_HH_1_, Homogeneity_HH_1_, Entropy_HH_1_, Variance_HH_1_	

#### 2.5.1 Grey-level co-occurrence matrix features

The Grey-level Co-occurrence Matrix (GLCM) is a second-order statistical matrix-based texture analysis method that was first proposed by (Haralick et al., 1973) to describe local heterogeneity information in images. This method quantifies the relationship between grey levels in an image by counting the pairs of pixels separated by a predefined distance (d) and direction (θ) that have the same distribution of grey-level values. Each pixel of the resulting matrix represents the number of times that the grey level of a reference pixel and the grey level of the neighbour pixel in the predefined distance d and direction θ are seen in the image. Consequently, the size of the GLCM will be Ng × Ng, being Ng the number of grey levels of the image. Several statistics representing the homogeneity or heterogeneity of the image can be mathematically computed from the GLCM.

In our study, images were uniformly quantised to Ng = 32 grey levels to reduce the computational cost of the feature extraction process and to improve the signal-to-noise ratio (Gibbs and Turnbull, 2003). A distance of d = 1 pixel was chosen to enhance mainly the local properties when computing the GLCM. To achieve rotation invariance, the features extracted from the GLCMs in the four directions of the 2D space (θ = 0°, 45°, 90° and 135°) were averaged. Rotation invariance is important in the context of our work because some texture methods like GLCM are dependent on the direction and different texture values could be obtained if the image is rotated, thus affecting the results when images from different patients have different orientations (Larroza et al., 2016). At the end, 13 texture features were extracted from the GLCM of each image, as shown in Table 1. The equations to compute these features and more details can be found in (Haralick et al., 1973).

#### 2.5.2 Grey-Level Run-Length Matrix features

The Grey-Level Run-Length Matrix (GLRLM) is also a statistical matrix-based texture analysis method of a higher-order than the GLCM that describes regional heterogeneity information. This method, first proposed by (Galloway, 1975) and extended by (Chu et al., 1990) and (Dasarathy and Holder, 1991), examines the times that each grey level value is seen consecutively in an image in a predefined direction. The GLRLM is constructed by detecting and counting the runs (sequences of consecutive pixels with the same grey level) of different grey levels and lengths in the image. Each row of the GLRLM represents a grey level and each column represents a specific length, so each pixel of the matrix indicates the number of runs of a specific grey level and length in the image. The features extracted from the GLRLM can be used to define fine textures (dominated by short runs) or coarse textures (dominated by longer runs) (Nailon, 2010).

To compute the GLRLMs, images were previously quantised to Ng = 32 grey levels as in the case of GLCMs. The GLRLM features are also affected by the orientation of the image, so features extracted from the GLCMs in the four directions of the 2D space (θ = 0°, 45°, 90° and 135°) were averaged to achieve rotation invariance. A total of 11 GLRLM features were computed, as shown in Table 1, and further details can be found in (Chu et al., 1990; Dasarathy and Holder, 1991; Galloway, 1975).

#### 2.5.3 Local Binary Patterns features

Local Binary Patterns (LBP) were introduced in (Ojala et al., 2002) and soon became very popular due to their high discrimination efficiency and computational simplicity. The LBP labels each pixel of the image under analysis by comparing its grey level with the grey levels of the surrounding pixels and then assigning a specific binary number. This binary number for each pixel is obtained by allocating a value of 1 to those surrounding pixels with a greater grey level value and a 0 to those surrounding pixels with a lower grey level value. Originally, LBP was defined for patches of 3 × 3 pixels, but it was later extended for blocks of *P* surrounding pixels separated by a distance *R*. Taking this generalization into account and given a pixel *c* with coordinates (x*_c_*,y*_c_*), the LBP binary number assigned to each pixel of the image is calculated using Eq. (1):

(1) $$LBP_{R,P}=\sum_{p=0}^{P-1}s\left(g_{p}-g_{c}\right)\times 2^{p}$$

where *g_c_* and *g_p_* are the grey level values of the central pixel *c* and its neighbour pixel *p*, and the function *s(g_p_ – g_c_)* is defined as:

(2) $$s\left(g_{p}-g_{c}\right)=\left\{\begin{array}{c} \;1\;if\;g_{p}-g_{c}\geq 0\\ 0\;if\;g_{p}-g_{c}<0 \end{array}\right)$$

Once the Eq. (1) is applied to all the pixels in the image, a LBP image is obtained and all the bins of the histogram of this image are used as texture features. Other statistics can be extracted from the LBP image and used as texture features like the mean or the variance.

In this work, the original LBP operator (patches of 3 × 3 pixels: *P* =  8, *R* = 1) was employed to preserve the texture analysis as local as possible because regions like WMH are not very large. Rotation invariance was achieved by performing a circular bit-wise right shift operation (rotating the neighbour pixel set clockwise) and assigning the smallest LBP binary number (Ojala et al., 2002). Using this approach, 36 unique rotation invariant histogram-based LBP features were obtained, as only 36 LBP binary numbers can occur for *P* =  8. Additionally, 4 statistics derived directly from the LBP image (median, variance, skewness and kurtosis) were added to the LBP features set. The MR images were not quantised to compute the LBP texture feature since the rotation invariant LBP approach is robust to intensity variations (Unay et al., 2007).

#### 2.5.4 Features based on the Wavelet transform

**Fig. 3:**
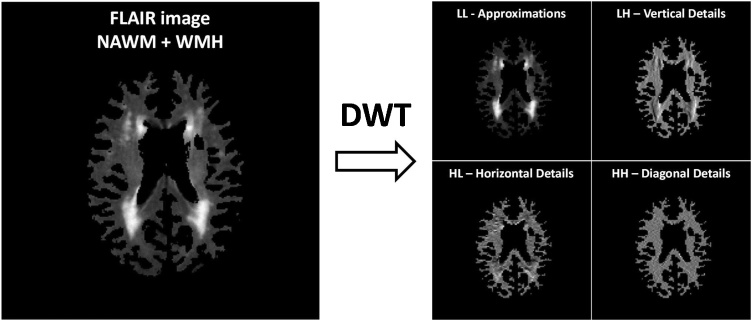
First DWT level of decomposition of a FLAIR image of the white matter tissue (NAWM and WMH) of a single brain slice.

The discrete Wavelet transform (DWT) is a technique that examines the spatial frequency patterns of an image within different scales and frequency directions, considering that frequency is directly proportional to grey level variations in an image. The DWT applied to an image produces four matrices of coefficients (sub-images) that represent the approximations or low frequencies (LL: low-low) and the details or high frequencies in the vertical (LH: low-high), horizontal (HL: high-low) and diagonal (HH: high-high) directions. An example of this matrices is shown in Fig. 3. The DWT can be repeated consecutively to achieve a major image decomposition: the first level of decomposition (LL_1_, LH_1_, HL_1_ and HH_1_) is applied to the original image as mentioned before and the subsequent levels are applied to the matrix of approximations of the previous level (LL*_i_*, LH*_i_*, HL*_i_* and HH*_i_*, where *i* is the level of decomposition). The DWT can be used as a transform texture analysis method by processing these sub-images to obtain parameters that describe the spatial frequency information of the image. These parameters have been previously used in some studies with successful results (Alegre et al., 2012; Arivazhagan and Ganesan, 2003; González-Castro et al., 2017).

In this work we examined two groups of texture features derived from the DWT. The first group was the Wavelet statistical features (WSF), consisting of 26 descriptors that are the mean and SD of the histograms of the original image and the sub-images yielded after three levels of decomposition. The second group was the Wavelet co-occurrence features (WCF), consisting of 24 descriptors that are obtained by extracting six of the GLCM features (energy, contrast, correlation, homogeneity, entropy and variance) from the sub-images yielded after the first DWT decomposition. The Haar family of wavelets was used to perform the DWT decomposition.

### 2.6 Statistical analysis and classification approach

#### 2.6.1 Evaluation of the suitability of the textural features

A preliminary statistical analysis was conducted to evaluate the discriminative power of each feature independently between populations. Its final purpose was to assess the feasibility of these features individually as biomarkers of stroke. To compare the distributions of each textural feature for each of the classes, the Mann-Whitney *U* test was applied. This non-parametric test is equivalent to the independent samples *t*-test but without the requirement of the normality assumption and is recommended for relatively small sample sizes (McDonald, 2014). As the number of statistical tests performed increases, the contrast test becomes more permissive, thus rejecting the null hypothesis more easily and increasing the number of false positives (McDonald, 2014). To counter this effect, usually known as the multiple comparisons problem, we decided to apply the Holm-Bonferroni correction before assuming the statistical significance of the features. This relatively strong (conservative) method controls the family-wise error rate, thus compensating the Type I error (incorrectly rejecting the null hypothesis) and attempting to limit the probability of even one false discovery.

#### 2.6.2 Classification models

After the statistical analysis, we applied machine learning algorithms to analyse the groups of texture features without removing any feature initially, since features that may be completely useless by themselves can provide a significant performance improvement when combined with others within a machine learning approach (Guyon and Elisseeff, 2003). We evaluated the performance of two well-known conventional classifiers: Support Vector Machine (SVM) with linear kernel and Random Forest (RF). The SVM classifier (James et al., 2013; S. Wang and Summers, 2012; Wu et al., 2008), in a binary classification task like ours, tries to maximize the margin distance between the classification boundary (i.e. hyperplane) and the closest samples of both classes by adjusting internal parameters in the training process. One of these parameters is the cost *C*, which controls the trade-off between misclassification of the training data and the size of the margins. Values of *C* = 2^−3^, 2^-2^, 2^-1^, 1, 2, 2^2^ and 2^3^ were tuned to obtain the optimal classification results. We used a linear kernel after an initial evaluation where non-linear kernels did not produce notably better results even after a lengthy training process. The RF classifier (Fernández-Delgado et al., 2014), combines the results of a multitude of independent and decorrelated decision trees in the training process, thus improving generalization of the model and robustness against overfitting in small samples (i.e. our sample is small for machine-learning algorithms). One of the advantages of the RF model is the little parameter tuning required. The parameter *mtry*, which identifies the number of random variables used in each tree, controls the strength (how accurate the individual trees are) and the correlation (the dependence between trees) of the RF model. Another tuning parameter is the number of trees to be built. In this work, values of *mtry* = 2, 4, 6, 8, 10 and 12 were evaluated and the number of trees was set to 250, as higher values of this parameter did no produce notably better results on a preliminary evaluation.

#### 2.6.3 Evaluation of the classification models

For evaluating the efficiency of the classification models, we employed a 5-fold cross-validation (CV) approach. This resampling method randomly partitions each texture dataset into five equally sized subsets of samples or folds, maintaining a balanced amount of both classes in each fold. Then, five models were trained and tested so that each of the five folds was used once as the test set, while the four remaining folds were used to train the model. This process was repeated ten times to reduce the variance of the cross validation results and to avoid possible bias in the random separation of the folds (Kuhn and Johnson, 2013c), so at the end 50 models (5 test folds × 10 repetitions) were built using different sets of patients for training and testing each time. The classification performance was evaluated using the averaged area under the curve (AUC) of the receiver operating characteristic (ROC) that resulted from averaging the AUC values obtained from the 50 iterations (mean ± SD). Good estimates of the model performance can be obtained using the validation data when the sample size is not large (Kuhn and Johnson, 2013c). Other metrics like sensitivity, specificity and accuracy were also obtained to validate the results.

The 9000 texture combinations (18 × 100 sets of images × 5 texture analysis methods) were firstly examined with the classifiers without excluding any texture feature, as previously mentioned. However, the texture combinations that provided the highest AUC values were analysed again using the same cross validation structure with a feature selection step included within the model-building process to avoid overfitting (Ambroise and McLachlan, 2002). This way, we could test if reducing the number of features improved the classification results. Two filter feature selection methods were applied to obtain rankings of features based on the discriminative power of each feature independently without analysing the relation between features and without involving any predictive model (Kuhn and Johnson, 2013a). The first method used the *p*-value provided by the Mann-Whitney *U* test for independent groups of samples. The second method used the Maximal Information Coefficient (MIC), which measures the strength of the linear or non-linear association between two variables.

**Fig. 4:**
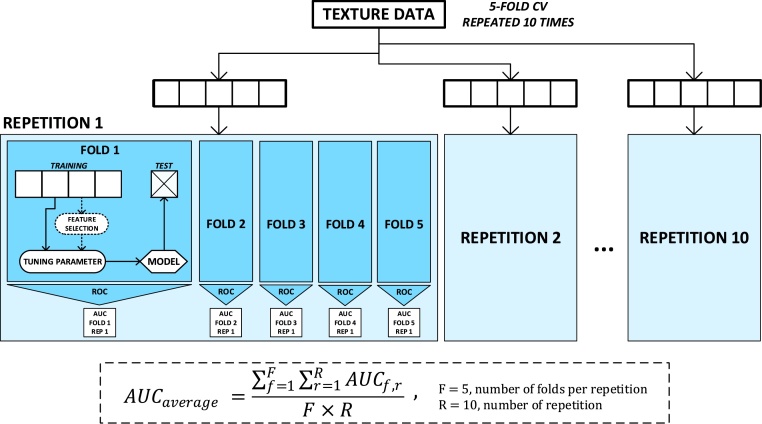
Cross-validation structure used to evaluate the 90 texture datasets. All the samples of each texture dataset were randomly separated R = 10 times in F = 5 folds to evaluate the model with the averaged AUC. This process was repeated for the two models studied (SVM with linear kernel and RF) and for all the chosen tuning parameters. The feature selection process was only applied to those sets which provided the best results to examine the influence of the number of features used to train the model.

The model evaluation process was implemented with the Caret package (Kuhn, 2008) in R language, version 3.2.5 (R Development Core Team, Vienna, Austria), and illustrated in Fig. 4.

#### 2.6.4 Influence of clinical indicators on the classifiers’ performance

The patients for which the best models performed well 80% or more of the times were identified. From these best models, the patients for which both classifiers (i.e. SVM and RF) performed well 80% or more of the times, were also identified. For each patient, we extracted the following data: 1) proportion of times the images were correctly (and wrongly) classified, 2) proportion of times in which both classifiers correctly (and incorrectly) classified the images using the same descriptors, 3) clinical stroke classification into no stroke, large cortical, small cortical or lacunar, 4) age at the time of scanning, 5) general WMH factor considering visual scores and computational measurements, as per (Aribisala et al., 2014), 6) percentage of lesion and normal tissue volumes in intracranial volume, 7) percentage of brain tissue volume in intracranial volume, 9) perivascular spaces visual scores in the basal ganglia and in the centrum semiovale. We calculated the bootstrapped bivariate Pearson’s correlations between the first two variables (i.e. results from the classification processes) and the rest (i.e. clinically derived variables) to evaluate possible influence of the clinical biomarkers and age in the outcome of the classification schemes.

## 3 Results

### 3.1 Textural features to discern between cortical and lacunar stroke patients

Sixty-one texture features of a total of 1026 features (114 features × 3 MRI sequences × 3 brain tissues/structures) were statistically significantly different (*p* < 0.05) between post-acute cortical and lacunar stroke patients, but only two features derived from the GLCM (FIMC and SIMC (see Table 1), with *p* =  0.0218 and *p* = 0.0096 respectively) were significant after applying a Holm-Bonferroni correction for multiple comparisons. Table 2 shows the distribution of significant features according to the MRI sequence and the brain tissue/structure. T1W sequences seem to be more suitable for using texture information to discriminate between cortical and lacunar stroke patients, especially when analysing the brain subcortical structures. Nevertheless, the texture data extracted from these images and these brain tissues/structures did not seem to have enough discriminative power to differentiate cortical and lacunar stroke patients in general.

**Table 2:** Number of significant features (*p* <  0.05) for discriminating cortical vs. lacunar stroke patients before (numerator) and after (denominator) Holm-Bonferroni correction for multiple comparisons per MRI sequence and brain tissue/structure.

MRI Sequence	Tissue or Structure		
	Normal-appearing white matter	Subcortical structures	White matter hyperintensities
FLAIR	0 / 0	16 / 0	1 / 0
T2W	3 / 0	9 / 0	1 / 0
T1W	11 / 0	19 / 2	1 / 0

Table 3 shows the averaged AUC (mean ± SD) computed from the 50 iterations when examining all the texture datasets with the two models under analysis (SVM with linear kernel and RF), and for all the MRI sequences and brain tissues/structures. A relevant AUC value could not be obtained (AUC < 0.7) in any case. The best result was obtained using the GLCM parameters in the T2W images of the NAWM region using SVM with linear kernel (AUC = 0.667 ± 0.117), but this value was not accurate enough to determine that a good classification can be achieved using these data.

**Table 3:** Results of the classification analysis for cortical and lacunar stroke patients. The AUC values computed by averaging the results of the validation data (mean ± standard deviation) are shown for the two models (SVM with linear kernel and RF) and for all the MRI sequences and brain tissues/structures when using the texture features extracted from the 5 texture analysis methods. The presented values are obtained for the best tuning parameter in each case.

AUC: Mean (SD)		GLRLM			GLCM			LBP			WCF			WSF		
		NAWM	SS	WMH	NAWM	SS	WMH	NAWM	SS	WMH	NAWM	SS	WMH	NAWM	SS	WMH
FLAIR	RF	<0.6	<0.6	<0.6	<0.6	<0.6	<0.6	<0.5	<0.6	<0.6	<0.5	0.600 (0.118)	<0.5	<0.5	<0.5	<0.5
	SVM	<0.6	<0.6	<0.5	<0.5	<0.6	<0.5	<0.6	0.604 (0.121)	<0.6	<0.6	<0.5	<0.5	<0.6	<0.5	<0.5
T2W	RF	<0.6	<0.6	<0.6	<0.6	0.611 (0.121)	<0.5	<0.5	<0.5	0.616 (0.107)	<0.5	<0.6	<0.6	<0.4	0.605 (0.117)	<0.4
	SVM	<0.5	0.622 (0.125)	<0.6	0.667 (0.117)	<0.5	<0.5	<0.6	<0.5	<0.6	<0.6	0.604 (0.129)	<0.6	0.621 (0.140)	<0.6	0.661 (0.132)
T1W	RF	<0.5	<0.5	<0.5	<0.6	<0.6	<0.6	<0.6	<0.6	<0.5	<0.6	<0.6	<0.5	0.649 (0.114)	<0.6	<0.5
	SVM	<0.5	<0.5	<0.6	<0.6	0.637 (0.140)	<0.6	<0.6	<0.6	0.616 (0.092)	<0.6	<0.5	<0.5	0.618 (0.128)	<0.5	<0.6

AUC: area under the curve, RF: random forest classifier, SVM: support vector machine classifier, GLRM: grey–level run length matrix features, GLCM: grey-level co-occurrence matrix features, LBP: local binary patterns features, WCF: wavelet co-occurrence features, WSF: wavelet statistical features, NAWM: normal-appearing white matter, SS: subcortical structures, WMH: white matter hyperintensities.

### 3.2 Texture analysis to classify individuals with vs. without previous stroke

Many texture features showed capability for discriminating between “no stroke” and “old stroke” individuals. In this case, 349/1026 texture features (114 features × 3 MRI sequences × 3 brain tissues/structures) were statistically significant (*p* < 0.05) when applying a Mann-Whitney U test for independent groups of samples, and 235 features remained significant after applying a Holm-Bonferoni correction for multiple comparisons. Table 4 shows the distribution of significant features according to the MRI sequence and the brain tissue/structure. The information collected in this table indicates that the texture features extracted from the brain subcortical structures are more effective in discriminating between “no stroke” and “old stroke” individuals, regardless of the MRI sequence is used.

**Table 4:** Number of significant features (*p* <  0.05) for discriminating “no stroke” and “old stroke” individuals before (numerator) and after (denominator) Holm-Bonferoni correction for multiple comparisons per MRI sequence and brain tissue/structure.

MRI Sequence	Tissue or Structure		
	Normal-appearing white matter	Subcortical structures	White matter hyperintensities
FLAIR	5 / 1	79 / 71	30 / 9
T2W	1 / 0	79 / 72	34 / 3
T1W	20 / 4	71 / 66	30 / 9

As per the AUC values obtained, certain groups of textures allowed “no stroke” and “old stroke” individuals to be classified with a good degree of precision. Table 5 shows the averaged AUC (mean ± SD) obtained from the 50 iterations when examining all the texture datasets with the two models (SVM with linear kernel and RF), and for all the MRI sequences and brain tissues/structures. Good results (i.e. AUC > 0.7) were not achieved with all groups of textures. Local binary patterns features extracted from T2W and FLAIR images of the subcortical structures delivered good results, as expected from the previous statistical analysis (see Table 4). However, other feature datasets like GLRLM features extracted from FLAIR images of the WMH or WCF features extracted from FLAIR images of the NAWM provided satisfactory results although the previous statistical analysis was not very optimistic with features extracted from these groups of images (Table 4). It should be noted that parameters extracted from the T1W images as well as parameters derived from the GLCM did not provide AUC values higher than 0.7 in any case. The predictive model employed for classifying the patients influenced the results, but there was not a firm conclusion on which model was better as SVM worked better with some texture datasets and RF with others.

**Table 5:** Results of the classification analysis for “old stroke” and “no-stroke” individuals. The AUC values computed by averaging the results of the validation data (mean ± SD) are shown for the two models (SVM with linear kernel and RF) and for all the MRI sequences and brain tissues/structures when using the texture features extracted from the 5 texture analysis methods. The presented values are obtained for the best tuning parameter in each case.

*AUC: Mean (SD)*		GLRLM			GLCM			LBP			WCF			WSF		
		NAWM	SS	WMH	NAWM	SS	WMH	NAWM	SS	WMH	NAWM	SS	WMH	NAWM	SS	WMH
FLAIR	RF	<0.6	0.691 (0.109)	0.674 (0.108)	<0.6	0.612 (0.099)	0.608 (0.111)	<0.5	**0.742 (0.100)**	<0.6	**0.761 (0.097)**	0.647 (0.099)	**0.702 (0.108)**	0.669 (0.114)	0.635 (0.094)	<0.6
	SVM	<0.6	0.676 (0.097)	**0.770 (0.089)**	<0.5	0.666 (0.090)	0.614 (0.124)	<0.5	**0.751 (0.103)**	0.682 (0.136)	0.637 (0.121)	<0.6	<0.6	0.637 (0.137)	<0.6	<0.6
T2W	RF	<0.5	0.643 (0.099)	<0.6	<0.5	0.641 (0.107)	0.617 (0.102)	<0.5	0.680 (0.112)	0.608 (0.116)	<0.5	0.680 (0.097)	**0.752 (0.097)**	<0.5	**0.705 (0.116)**	0.665 (0.103)
	SVM	0.665 (0.084)	**0.738 (0.121)**	0.646 (0.128)	0.601 (0.138)	0.644 (0.111)	<0.5	<0.6	**0.763 (0.116)**	0.671 (0.122)	<0.5	0.608 (0.157)	<0.5	<0.6	**0.737 (0.103)**	0.677 (0.123)
T1W	RF	0.609 (0.104)	0.654 (0.112)	<0.6	<0.6	0.662 (0.091)	0.659 (0.113)	0.667 (0.125)	0.649 (0.120)	0.611 (0.140)	0.624 (0.105)	0.682 (0.109)	0.664 (0.115)	<0.6	<0.5	<0.6
	SVM	<0.6	0.662 (0.104)	<0.5	0.642 (0.126)	<0.6	<0.5	<0.6	0.676 (0.122)	0.630 (0.126)	0.628 (0.143)	<0.6	<0.6	<0.6	<0.5	<0.6

^*^Values in bold indicate the best AUC results (AUC > 0.7).

AUC: area under the curve, RF: random forest classifier, SVM: support vector machine classifier, GLRM: grey–level run length matrix features, GLCM: grey-level co-occurrence matrix features, LBP: local binary patterns features, WCF: wavelet co-occurrence features, WSF: wavelet statistical features, NAWM: normal-appearing white matter, SS: subcortical structures, WMH: white matter hyperintensities.

#### 3.2.1 Influence of the feature selection on the classification results

**Fig. 5:**
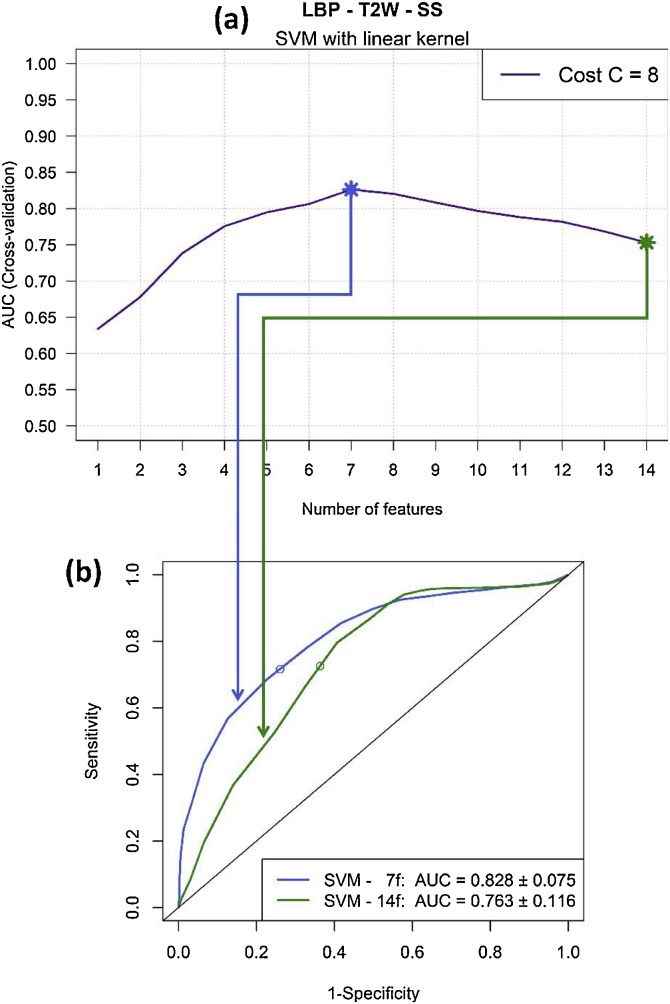
Results of applying the feature selection method based on MIC to the texture dataset of LBP features extracted from T2W images of subcortical structures (SS) when training the SVM model with cost C = 8. The profile of AUC values obtained for all possible subsets of features according to the MIC ranking is illustrated in (a). The ROC curves provided by the model when using all the features (14 features) and when using the optimal number of features (7 features) is shown in (b).

We applied two filter feature selection methods to the five texture datasets that yielded good results (i.e. AUC > 0.7) to see if classification results improve when reducing the number of features. Rankings of features based on the Maximal Information Coefficient (MIC) and the *p*-value provided by the Mann-Whitney *U* test were computed from the training folds in each of the 50 iterations of the CV procedure. Table 6 shows the new AUC values obtained when reducing the number of features according to the computed rankings in the best texture datasets. In general, better AUC values were obtained when reducing the number of features. In particular, it is remarkable the substantial improvement achieved for LBP parameters extracted from T2W images of the subcortical structures when using the SVM model: a final value of AUC = 0.828 ± 0.075 was obtained when only using the 7 more relevant LBP characteristics according to the MIC coefficient. Fig. 5 shows the AUC values obtained for all possible subsets of features according to the MIC ranking and the ROC curves provided by the model when using all the features and when using the optimal number of features.

**Table 6:** Values of AUC obtained when analysing the best texture datasets with and without applying feature selection, i.e. using all the features of the dataset (All) and reducing the number of features based on two metrics: the *p*-value (*p*-val) and the maximal information coefficient (MIC).

*AUC: Mean (SD)*	GLRLM FLAIR – WMH			LBP FLAIR – SS			LBP T2W – SS			WCF FLAIR – NAWM			WCF T2W – WMH		
	All	*p*-val	MIC	All	*p*-val	MIC	All	*p*-val	MIC	All	*p*-val	MIC	All	*p*-val	MIC
RF	0.674 (0.108)	=*	=	0.742 (0.100)	=	0.744 (0.104)	0.680 (0.112)	0.693 (0.101)	0.714 (0.113)	0.761 (0.097)	0.766 (0.099)	0.766 (0.086)	0.752 (0.097)	=	=
SVM	0.770 (0.089)	0.773 (0.089)	0.773 (0.093)	0.751 (0.103)	=	0.759 (0.103)	0.763 (0.116)	0.774 (0.099)	0.828 (0.075)	0.637 (0.121)	0.713 (0.125)	0.712 (0.112)	<0.5	<0.6	=

AUC: area under the curve, RF: random forest classifier, SVM: support vector machine classifier, GLRM: grey–level run length matrix features, GLCM: grey-level co-occurrence matrix features, LBP: local binary patterns features, WCF: wavelet co-occurrence features, WSF: wavelet statistical features, NAWM: normal-appearing white matter, SS: subcortical structures, WMH: white matter hyperintensities, MIC: maximal information coefficient.

* The symbol “=” is used when no improvement is obtained by reducing the number of features.

#### 3.2.2 Influence of age in the classification results

Table 7 shows the results obtained with and without including the image data from the older individuals (i.e. age ˜91 years old) in the texture datasets that performed better (i.e. AUC > 0.7) in the previous analysis. The results show that the classification performance got worse when introducing older patients in general, suggesting that age influences the classification results by increasing the misclassification rate.

**Table 7:** Values of AUC obtained when analysing the best texture datasets (without feature selection) with and without including the textures extracted from the additional older patients.

*AUC: Mean (SD)*	GLRLM FLAIR – WMH		LBP FLAIR – SS		LBP T2W – SS		WCF FLAIR – NAWM		WCF T2W – WMH	
	Without older patients	With older patients	Without older patients	With older patients	Without older patients	With older patients	Without older patients	With older patients	Without older patients	With older patients
RF	0.674 (0.108)	0.682 (0.089)^a^	0.742 (0.100)	0.655 (0.098)	0.680 (0.112)	0.623 (0.078)	0.761 (0.097)	0.645 (0.086)	0.752 (0.097)	0.678 (0.074)
SVM	0.770 (0.089)	0.736 (0.084)	0.751 (0.103)	0.644 (0.106)	0.763 (0.116)	0.670 (0.083)	0.637 (0.121)	0.580 (0.106)	<0.5	<0.5

AUC: area under the curve, RF: random forest classifier, SVM: support vector machine classifier, GLRM: grey–level run length matrix features, GLCM: grey-level co-occurrence matrix features, LBP: local binary patterns features, WCF: wavelet co-occurrence features, WSF: wavelet statistical features, NAWM: normal-appearing white matter, SS: subcortical structures, WMH: white matter hyperintensities.

a Exception where the AUC increased after adding older patients.

### 3.3 Clinical evaluation of the outcome of the models

None of the clinical biomarkers analysed correlated with the proportion of times the images were correctly (and wrongly) classified, and neither with the proportion of times in which both classifiers correctly (and incorrectly) classified the images using the same descriptors. Only age was significantly correlated with these measurements (*p* ≤ 0.001). However, the strength of the significance was only r = 0.31 and r = 0.39 respectively.

**Fig. 6:**
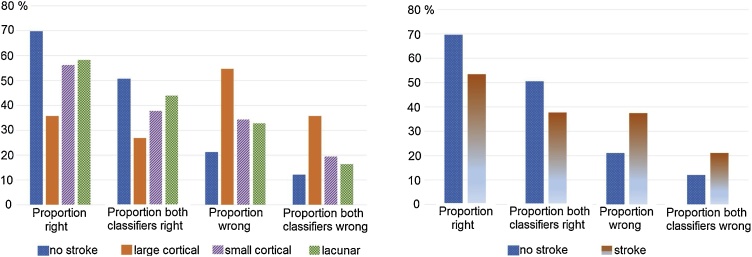
Pattern of the classification performance of the best models (i.e. for which the accuracy was above 80%) per stroke subtype (i.e. no stroke, large cortical, small cortical or lacunar) (left) and per stroke occurrence (i.e. had stroke or not) (right).

The pattern of the classification performance of the different types of images (i.e. no stroke, large cortical stroke, small cortical stroke, lacunar stroke) was similar irrespective of the classifier used (i.e. SVM and RF) and when the analysis accounted for whether the images were correctly (or incorrectly) classified by both classifiers: “no stroke” images achieved the greatest proportion of well classified, followed by “lacunar”, “small cortical” and “large cortical” (Fig. 6).

## 4 Discussion

In this paper, the performance of several texture features extracted from different brain tissues and structures (i.e. NAWM, WMH and subcortical structures) in different MRI structural sequences (i.e. FLAIR, T2W and T1W) was analysed, in two conventional machine learning approaches, to identify the presence of stroke in the images of post-acute stroke patients and normal ageing individuals. Differentiation of post-acute cortical vs. lacunar stroke subtypes using texture analysis was examined as well as classification of images with vs. without chronic stroke lesions.

Only two textural features from the subcortical structures in T1W images resulted with high discriminatory power between images from post-acute lacunar vs. cortical stroke. These features were the first and second information measures of correlation (FIMC and SIMC) derived from the GLCM, which quantify the linear dependency or correlation between intensities, thus representing homogeneity but adding some desirable properties that are not represented by the original correlation descriptor extracted from the GLCM (Haralick et al., 1973). It can be questioned the reason behind using all Haralick features in our models, given that some of them maybe correlated, carrying repetitive information. A review on feature selection methods (Guyon and Elisseeff, 2003) agrees that perfectly correlated variables are truly redundant in the sense that no additional information is gained by adding them, so the removal of perfect correlated variables will not negatively impact the learning performance. However, the authors also state that very high variable correlation (or anti-correlation) does not mean absence of variable complementarity: features that do not carry any discriminatory or added value by themselves can provide a significant improvement in performance when combined with others within a machine learning approach.

A previous study that evaluated the use of texture analysis as an alternative for characterising SVD and assessing possible blood brain barrier leakage (Valdés-Hernández et al., 2017) reported differences in the texture of the FLAIR deep grey matter between post-acute lacunar and cortical stroke patients, but only with borderline significance. That study reported the texture in the deep grey matter was more ‘homogeneous’ in patients with recent lacunar stroke compared to those who had a cortical type. Statistically significant differences between the FLAIR images from both groups of patients were only found in the textural features measured in the post-acute stroke lesions. Our motivation was to explore whether the texture in the conventionally segmented tissues (i.e. normal and abnormal) could have enough discriminatory power to be used in machine learning schemes to identify the stroke subtype and if there was a stroke. Usually ischaemic stroke lesions and artefacts that mimic WMH are included within the burden of WMH by automatic WMH segmentation methods. A real-world scenario would have been to evaluate the output of the-state-of-the-art CNN WMH segmentation methods. However, corruption of the abnormal WMH signal with uneven burden of signal changes from other causes would have introduced a bias in the results. Therefore, we carefully excluded the stroke lesions from the burden of hyperintense T2W-based signal and did not analyse the texture in them. Our analysis found features with borderline statistical significance to discriminate between cortical and lacunar stroke patients in T1W MRI data but failed to find a conventional machine learning model to classify these patients accurately. The reason behind these results may lie in the fact that both types of stroke can be seen simultaneously in many cases, as reported by (Xu, 2014).

The images from “no stroke” patients were, in general, better identified by the classifiers as opposed to the images that had “large cortical” chronic stroke lesions, which resulted in them being less well classified, and, instead, were classed as not having any stroke lesion at all. It might seem contradictorily, given that the images of individuals with chronic lacunar lesions (i.e. small lesions mainly in the region crossed by the corticospinal tracts (Valdés-Hernández et al., 2015a,b), which can be confounded by WMH, were the second best classified. However, lacunar, and not large cortical, strokes have been associated with blood brain barrier impairment, manifested in abnormal extracellular leakage (Wardlaw et al., 2017). Also, textural features in normal and abnormal tissues have been reported as being useful in detecting the subtle differences that this mechanism causes.

One limitation of this work is the impossibility of combining the stroke and ageing datasets to analyse if images showing recent cortical or lacunar strokes could be distinguished from images of patients without stroke and patients with an old stroke lesion. This is because variations in acquisition parameters may result in differences in the texture outcome that are not due to the underlying biological characteristics of the tissues expressed by the texture (Mayerhoefer et al., 2009; Schad, 2004). Image normalization techniques help to reduce differences in imaging acquisition settings, but some residual effects may not be totally suppressed, thus obscuring the true texture differences due to the tissue properties only. Therefore, combining texture features extracted from both databases in the machine learning pipeline evaluated may lead to overoptimistic results caused by the differences in imaging acquisition protocols. Other limitation of the present work consists on the 3D texture analysis approach based on the median of 2D texture features. Although pure 3D texture analysis is usually preferred because it allows capturing more heterogeneity information of the tissue under analysis, this approach is not always feasible, especially when the slice thickness of the images is very large compared to the in-plane resolution (Depeursinge et al., 2014), as in our case. In these situations, 2D texture approaches or approaches like the one carried out are recommended.

In this work we conducted a very detailed texture analysis study for identifying and characterizing ischaemic stroke lesions in structural brain MRI data by considering several regions or tissues and by testing a large amount of quantitative texture descriptors. The number of patients per group was sufficiently large to draw reliable conclusions and the machine learning pipeline was designed to avoid overoptimistic and overfitted results. We achieved promising results that suggested that texture features may help in the detection of previous stroke lesions, and identified the textural features that look promising on this task, so as they can be evaluated in transfer learning schemes with the-state-of-the-art deep CNNs. Additionally, the correlation of our results with clinical parameters was explored to find clinical patterns or characteristics that could be reflected in the textural features that resulted promising to the classification tasks evaluated.

## Conflicts of interest

All authors declare no Conflict of Interests to report.

## Study funding

This work was funded by the Row Fogo Charitable Trust (MVH, VGC) grant no. BRO-D.FID3668413, and the Wellcome Trust (patient recruitment, scanning, primary study Ref No. 088134/Z/09). The study was conducted independently of the funders who do not hold the data and did not participate in the study design or analyses.

The Lothian Birth Cohort 1936 is funded by Age UK (Disconnected Mind grant) and the Medical Research Council (MRC; MR/M01311/1, G1001245, 82800), and the latter supported BSA. IJD was supported by the Centre for Cognitive Ageing and Cognitive Epidemiology, which is funded by the MRC and the Biotechnology and Biological Sciences Research Council (MR/K026992/1).

David Moratal acknowledges financial support from the Spanish Ministerio de Economía y Competitividad (MINECO) and FEDER funds under Grant BFU2015-64380-C2-2-R, and from the Conselleria d'Educació, Investigació, Cultura i Esport, Generalitat Valenciana (grants AEST/2017/013 and AEST/2018/021).

Rafael Ortiz-Ramón was supported by grant ACIF/2015/078 and grant BEFPI/2017/004 from the Conselleria d’Educació, Investigació, Cultura i Esport of the Valencian Community (Spain).
